# The effect of fibromyalgia syndrome to gravidity, parity and duration of breastfeeding; A prospective study from Turkey

**DOI:** 10.12669/pjms.323.9574

**Published:** 2016

**Authors:** Koca Tuba Tulay, Tanrikut Emrullah, Arslan Aydin, Ozdemir Filiz Ciledag

**Affiliations:** 1 Koca Tuba Tülay, Medical Doctor, Dept. of Physical Medicine and Rehabilitation, State Hospital, Malatya, Turkey; 2 Tanrikut Emrullah, Operator Doctor, Dept. of Obstetrics and Gynecology, State Hospital, Malatya, Turkey; 3 Arslan Aydin, Operator Doctor, Dept. of Orthopaedics and Traumatology, State Hospital, Malatya, Turkey; 4 Ozdemir Filiz Ciledag, Physical Therapist, Department of Physical Medicine and Rehabilitation, Inonu University, Malatya, Turkey

**Keywords:** Pregnancy, Fibromyalgia, Breastfeeding

## Abstract

**Objectives:**

Fibromyalgia syndrome (FS) is a chronic pain disorder usually affecting women in their fertile period of life. However, the relationship between FS and pregnancy has not been studied in depth. The effect of FS on the course of pregnancy is poorly investigated in the current literature. Here we aimed to investigate the effects of FS to menarche age, gravidity, parity and duration of breastfeeding.

**Methods:**

One hundred and eighty-seven non-pregnant females attending between March 2015-June 2015, to Malatya State Hospital Physical Medicine and Rehabilitation Outpatient Clinic, were included in this prospective study. One hundred eleven (111) of them were diagnosed with FS according to the American Rheumatology Association (ACR) 2010 criteria and were defined as group 1; group 2 comprised of seventy six (76) non-pregnant healthy volunteers. All participants were asked about their menarche age, marriage age, gravidity, parity, duration of breastfeeding by conducting a basic questionnaire survey. Patients’ body mass index (BMI) were recorded. Depression parameters were evaluated by Beck Depression Scale (BDS).

**Results:**

The average age of the patients was 39.04±9.21 (FS) and 38.47±9.65 (Control) years; first menarche age was at 13.28±1.38 (FS) and 13.59±1.54 years (Control), and marriage age was 20.1±3.62 (FS) and 20.69±3.90 years (Control), respectively. No statistically significant difference was found (*p*=0.598) between BMI values (FS, 27.76±4.95; Control 26.90±4.56 kg/m^2^). The results from both groups were similar in terms of gravidity, parity, and breastfeeding duration, with no statistically significant differences (p=0.252, 0.093, 0.075, respectively). The only significant difference was found in the depression parameter. The BDS results were statistically different between the groups, found higher in FS group (p=0.000).

**Conclusion:**

FS occurs as a result of symptoms such as mood disorder, anxiety, cognitive and sleep disorders, and also hormonal changes; no exact cause has yet been established. The syndrome usually occurs during fertile period of young female. According to the findings of our study, FS has no negative effect on the outcome of gravidity, parity, and duration of breastfeeding in Turkish women. Further studies about the effects of FS on the course of pregnancy are required.

## INTRODUCTION

Fibromyalgia syndrome (FS) is a chronic disease involving a range of symptoms including diffuse pain, sleep disorder, and cognitive function disorder (usually depression).^1^,^2^ FS is usually seen in women between 30 and 50 years of age and its prevalence ranges between 1 up to 4%.^3^ Its etiology and pathogenesis are still not known, but it is thought to be a multifactorial disease. Neural activity changes in the central nervous system, abnormal metabolism of biogenic amines, and immunologic problems may lead to the occurrence of this disease. The subjectivity of FS narratives, which is mostly similar with chronic pain and psychosomatic symptoms, makes differential diagnosis problematic.^4^

FS is a chronic pain disorder that affects women of childbearing age, but the relationship between FS and pregnancy is not widely studied. However, FS symptoms affect pregnancy status, spontaneous abortion, menstruation, oral contraceptive usage, and breastfeeding duration.^5^

Neither the effect of FS on the course of pregnancy nor the converse is known, as a result of lack of studies. Here, we aimed to investigate the effects of FS to gravidity, parity and breastfeeding duration in Turkish patients.

## METHODS

One hundred and eleven (111) patients attending during March 2015 - June 2015, to Malatya State Hospital Physical Medicine and Rehabilitation Outpatient Clinic, diagnosed with FS according to 2010 ACR criteria^6^ were included in this prospective study. Non-pregnant patients were included the study. A simple questionnaire survey was conducted in all participants. İn survey, patients were asked about their number of previous pregnancy (gravidity), alive birth (parity), age at first menarche, age at marriage and duration of breastfeeding. Body mass index of patients was recorded. Depression parameters were evaluated by Beck Depression Scale (BDS). Control group comprised of 76 non-pregnant healthy volunteers. All patients were informed about the study, which was performed according to the terms of the Helsinki Declaration and received prior ethics committee approval.

Exclusion criteria were defined as inflammatory rheumatism disease, acute infection, malignancy, and co-occurring disease and pregnancy. The SPSS21 version was used for data analysis. Normal distribution of data was checked using the Kolmogorov–Smirnov test. For each parameter, a comparison was done between the two groups group. The *t*-test was used for normally distributed groups while the Mann–Whitney U-test was used for abnormal distribution. *P*<0.05 was evaluated as statistically significant.

## RESULTS

Total 187 participants (111 FS; 76 Control) were included in the study. The average age of the patients was 39.04±9.21 (FS) and 38.47±9.65 (Control) years; first menarche age was at 13.28±1.38 (FS) and 13.59±1.54 years (Control), and marriage age was at 20.1±3.62 (FS) and 20.69±3.90 years (Control), respectively. No statistically significant difference was found (*p*=0.598) between BMI values (FS, 27.76±4.95; Control 26.90±4.56 kg/m^2^). Gravidity for the FS group was 1–13 and for the control group was 1–7. Parity for the FS group was 1-8 and for the control group was 1–8. No statistically significant difference was found (*p*=0.252 and 0.093, respectively). The duration of breastfeeding in the FS and control groups was 36.20±22.19 months and 31.52±23.53 months, respectively. The Beck depression index (BDI) for the FS and control groups was 3:23, 4:31, 5:16, 6:1 and 1:49, 2:22, 3:2, 4:3, 1:17, 2:23, respectively. According to these results, the BDI was found to be higher in the FS group (*p*=0.000). The above results are summarized in Table-I.

**Table-I:** Main study findings.

	*Control group*	*Fibromyalgia group*	*P-value*
Number of patients	76	111	
Age (years)	38.47±9.65 (22–64)	39.04±9.21 (24–65)	0.574
Body mass index (BMI)	26.90±4.56	27.76±4.95	0.598
Age at menarche (years)	13.59±1.54 (9–17)	13.28±1.38 (10–17)	0.178
Age at marriage (years)	20.69±3.90 (14–37)	20.1±3.62 (13–33)	0.251
Number of pregnancies (gravidity)	2.93±1.49(1–7)	3.23±1.74 (1–13)	0.252
Number of births (parity)	2.51±1.31 (1–8)	2.76±1.27 (1–8)	0.093
Duration of breastfeeding (months)	31.52±23.53 (2–114)	36.20±22.19 (0–96)	0.075
Beck depression index (BDI)	Median:1, mode:1	Median:3, mode:4	0.000

## DISCUSSION

FS is a chronic pain disorder characterized by diffuse sensitivity and various somatic symptoms. Since publication of the ACR classification criteria, the number of studies investigating the etiology and long-term effects of the disease has risen. In 2010, new criteria were evaluated to simplify clinical diagnosis^6^,^7^; these were established as a result of insufficient evaluation of the 1990 criteria. Here, we diagnosed FM using the 2010 criteria.

FS is especially seen in women of childbearing age and it negatively affects the quality of life. Additionally, it can affect the number of pregnancies, course of pregnancy and duration of breastfeeding in terms of hormonal imbalance, cognitive disorders, negative effects on sleep quality, and mood disorders such as depression following anxiety. There is a lack of scientific researches on this topic.

FS symptoms are usually associated with a minimum of changes in the three central nervous system: (1) chronic locomotor pain, indicating disruption of the pain processing centers; (2) anxiety; and (3) depression. The role of sex hormones in pain mechanisms and their various effects on pain receptors need to be understood to reveal the processes resulting in chronic pain syndromes. The relationship between FS and hormonal imbalance in middle age demonstrates the importance of sex hormones in the development of FS. However, there is insufficient evidence from the existing literature to support this idea. However, it was shown that pain episodes were decreased by hormone-mediated modulation of the pain pathways with adrenal, thyroid, and ovarian hormone.^8^,^9^

An increase in levels of basal adrenocorticotropic hormone (ACTH), follicle-stimulating hormone (FSH) and cortisol; a decrease in the levels of insulin-like growth hormone1 (IGF-1, somatostatin C), free triiodothyronine (FT3) and estrogen were detected. Systemic increase in corticotropic hormone (CRH), growth hormone-releasing hormone (GHRH), thyrotropin-releasing hormone (TRH), and luteinizing hormone-releasing hormone (LHRH) resulted in an increase in ACTH and prolactin (PRL) levels and a decrease in thyroid-stimulating hormone (TSH). Hormonal changes in FS patients can be explained by chronic pain-dependent–primary stress activation of hypothalamic CRH neurons.^8^-^10^

FS is mostly seen in women rather than men, with a concomitant role of disrupted sex hormones. Okifiju and colleagues^11^ compared sex hormone levels and pain sensibility at different menstrual cycle stages in FS patients and a control group. According to this study, an increase in substance P levels at mid-luteal phase was detected in FS patients relative to their healthy coevals. Additionally, pain threshold and pain tolerance during the menstrual cycle of FS patients were found to be lower than in the control group. Taking these findings into account, the high frequency of FS disease is not only due to hormonal disruption, but is also related to pain sensitivity; however, the role of sex hormones in hyperalgesia remains to be determined. Schochat et al.^12^ found a correlation between FS, late menarche, and decreased fertility. In our study, there was no statistically difference between the FS and control groups in terms of age at menarche, age at marriage, gravidity and parity. Pain complaints during the menstrual cycle were not requested as educational level of our patients was mostly elementary and primary school. Also they couldn’t gave enough information about their beginning time of fibromyalgia symptoms and their previous course of pregnancies particularly (delivery complications, abortions etc).

As a disease of childbearing age, the effect of FS on the course of pregnancy has not been determined due to the inadequate level of research yet. The symptoms of FS show an increased profile in healthy pregnant women near their third trimester.^13^-^15^ The lordosis which occurs due to balance the growing uterus shifts back the center of gravity on upper extremity. One interesting anthropological study shows that this lumbar vertebral support is used to maintain moving ability and bipedal posture against maternal weight increase by 31% in human.^14^ During pregnancy, an increase is observed in sacroiliac, sacrococcygeal and pubic joint mobility. This may create problems in regard to maternal postural changes and especially, may lead to back and belly pain in later pregnancy,^13^-^16^ along with pain, insensitivity, and weakness. This situation can also arise with the occurrence of distinct lordosis and shoulder collapse following ulnar and median nerve traction.^13^ The occurrence of this situation may affect perinatal period and anesthesia requirements. İn another study, Ostensen et al.^5^ showed that the symptoms of FS were exacerbated by pregnancy, with the last trimester being the worst period. Additionally, they noted that postpartum depression and anxiety were the most distinct patient’ complaints and no effect of FS was observed on pregnancy and the health of the newborn.^5^ In the study of Leblebici et al.^17^, a correlation between pregnancy and FS was found in the geriatric group. It is known that estrogen levels can affect the pathogenesis of this disease, but to date no study has shown a correlation between estrogen levels and disease.^18^

We did not request about their course of pregnancy in this study as we did not include pregnant women and our patients couldn’t gave correct information about their previous pregnancies. We detected no difference between two groups in terms of number of pregnancies; by taking this into account, we can conclude that FS does not have any negative effect on gravidity, parity and duration of breastfeeding in Turkish patients. As a result we couldn’t say FS totally has no effect to pregnancy and breastfeeding, because there are so many factors which affect pregnancy. It may be expected that duration of breastfeeding would be shorter in mothers with FS. The Turkish cultural encouragement of breastfeeding and giving birth may have some effect in this regard. How does FS affect the duration of breastfeeding? During lactation, it is possible that incorrect breastfeeding positions (neck protrusion or leaning over the baby) may lead to chronic muscle pains by creating micro-traumas in the muscles and tension in nerves and ligaments.^13^ Also chronic pain, muscle rigidity, sleep and mood disorders contribute it. Schafer KM et al.^19^ evaluated breastfeeding experience in mothers with FS, and observed that some mothers felt they were incapable of breastfeeding. Especially, muscle aches were found to be the biggest problem with tiredness, depression, and mood disorders also negatively affecting breastfeeding. In the present study, depression was seen more in FS patients according to the BDI. Therefore, it is recommended that this population needs to be well informed, emotionally and physically supported.^19^ The treatment approach should be on an individual basis because of the heterogeneity of FS in terms of pain severity, presence of other symptoms, comorbidity, level of functional incapability. In addition, the control of the disease should include the definition of all comorbid pain sources (peripheral and visceral pain) and treatments. Consequently, it is desirable to treat patient’s overall functioning and health.^20^,^21^ We should not forget to include central-meditative pain when we formulate the treatment for FS patients.^10^

FS treatment is long-term and complex, antidepressants and physiotherapy play a major role. A multi-modal approach, including cognitive-behavior education programs such as stress supervision, pain control, and relaxing techniques, is suggested to be effective.^22^ There is considerable discussion among clinicians about the control of chronic pain during lactation, and some FS patients could not be treated on account of medication phobia. Some analgesics are completely contraindicated during pregnancy and breastfeeding, but the pain medication data in pregnant women is not enough to make a conclusion.^14^-^16^

Duloxetine hydrochloride is commonly used in the treatment of major depressive disorder, common anxiety disorder, diabetic periphery neuropathy, chronic muscle skeleton pain, and FS. These diseases are usually seen mostly in women of childbearing age. Mothers should decide whether to use medication during pregnancy by considering its effects on the fetus. However, there is inadequate research on Duloxetine use by pregnant women. In a 2011 study of the FDA side effects reporting system database, Hoog et al.^23^ found that the side effects of Duloxetine usage in pregnant women were similar to those in the general population. Our patients stated that they did not use medication because of chronic pain during their pregnancy: they used only nonsteroidal anti-inflammatory medications, irregularly.

FS-like symptoms such as muscle pain and sensitivity, burnout syndrome, decreased exercise capacity, and cold intolerance show similar symptoms to endocrine dysfunctions such as hypothyroid, adrenal, and growth hormone deficiency. Some controlled clinical studies show that hormone supplements may be beneficial for FS patients with no serious hormone abnormality.^24^

## CONCLUSIONS

FS is a common condition in women of childbearing age and negatively affects the quality of life. A wide range of symptoms and findings demonstrate that it may affect both course of pregnancy and breastfeeding. We found no negative effect of FS on gravidity, parity and duration of breastfeeding in Turkish patients. Depression was reported more frequently by patients with FS than the control group. Further studies are needed to confirm our observations.
